# The cecal and fecal microbiomes and metabolomes of horses before and after metronidazole administration

**DOI:** 10.1371/journal.pone.0232905

**Published:** 2020-05-22

**Authors:** Carolyn E. Arnold, Anitha Isaiah, Rachel Pilla, Jonathan Lidbury, Josie S. Coverdale, Todd R. Callaway, Sara D. Lawhon, Joerg Steiner, Jan S. Suchodolski

**Affiliations:** 1 Department of Large Animal Clinical Sciences, College of Veterinary Medicine & Biomedical Sciences, Texas A&M University, College Station, Texas, United States of America; 2 Gastrointestinal Laboratory, Department of Small Animal Clinical Sciences, College of Veterinary Medicine & Biomedical Sciences, Texas A&M University, College Station, Texas, United States of America; 3 Department of Animal Science, Texas A&M University, College Station, Texas, United States of America; 4 Department of Animal and Dairy Science, University of Georgia, Athens, Georgia, United States of America; 5 Department of Veterinary Pathobiology, College of Veterinary Medicine & Biomedical Sciences, Texas A&M University, College Station, Texas, United States of America; Queen's University Belfast, UNITED KINGDOM

## Abstract

Antibiotic administration can be a cause of gastrointestinal disease in horses, creating a disruption in the normal population and function of bacteria found in the hindgut. The objective of this study was to describe the changes in the cecal and fecal microbiomes and metabolomes of clinically healthy horses before and after metronidazole administration. Metronidazole (15 mg/kg BID PO) was given to five horses with cecal cannulas. The study was suspended on Day 3 due to adverse gastrointestinal effects. Cecal and fecal samples were obtained before (Days minus52, m28, m14, and 0) and after (Days 7, 14, 28, and 52) metronidazole administration. DNA was extracted from the cecal and fecal samples, and 16S rRNA genes were sequenced. Richness and evenness indices were significantly decreased by metronidazole administration in both cecal and fecal samples, but the overall composition was only significantly changed in fecal samples on Day 3 (ANOSIM, p = 0.008). The most dominant phyla were Bacteroidetes and Firmicutes in all groups examined. In fecal samples, significant changes of the phyla Actinobacteria, Spirochaetes, Lentisphaerae, and Verrucomicrobia occurred on Day 3, which correlated with clinical signs of gastrointestinal disease. The metabolome was characterized by mass spectrometry-based methods and only named metabolites were included in the analysis. Fecal, but not cecal, metabolites were significantly affected by metronidazole. The fecal metabolites affected represent diverse metabolic pathways, such as the metabolism of amino acids, carbohydrates, lipids, nucleic acids and cofactors and vitamins. Metronidazole administration has potential to cause adverse effects in horses, alters the bacterial composition of the horse’s cecal and fecal content, and the metabolome of fecal samples.

## Introduction

Amongst veterinary species, horses have a comparatively high degree of morbidity and mortality attributed to diseases of their gastrointestinal tract (GIT) [1]. An emerging body of evidence links GIT disease, such as colic and colitis, to alterations in the bacterial communities in the equine hindgut [2–5]. The term dysbiosis has been used to describe the changes in the microbial communities associated with disease states in other species [6, 7], a concept which appears relevant for the horse. While it is not always clear if dysbiosis is the cause or effect of disease, it is well established that the resident microbiota play a vital role in the health of the horse by providing energy and nutrient needs via the fermentation of plant products [2, 4, 5, 8]. For horses with colitis, the physiologic consequences of dysbiosis potentially include an overgrowth of pathogenic bacteria, bacterial toxin production, mucosal inflammation, alterations in the metabolism of carbohydrates, volatile fatty acids and bile acids [9].

Equine colitis is typically classified by etiology (e.g., infectious, antimicrobial-associated, non-steroidal anti-inflammatory-induced, inflammatory infiltrates, unknown, etc.) [10]. To date, there are few studies describing the microbial community structure associated with the various types of colitis. Antibiotic associated diarrhea (AAD) is a common sequelae of antimicrobial administration, and is associated with a higher risk of mortality than other forms of colitis [11]. Antimicrobial agents, which are commonly administered to horses to treat established infections and to provide prophylaxis for surgically created wounds, affect the microbiome of the hindgut of the horse. Currently, tetracyclines, macrolides, cephalosporins, fluoroquinolones, trimethoprim-sulphonamides, aminoglycosides, chloramphenicol, and β-lactams all have been reported to cause colitis in horses [3, 12].

Paradoxically, some antimicrobial agents, such as metronidazole, have been found useful in the treatment of colitis. Metronidazole is a bactericidal nitroimidazole with activity against anaerobic bacteria and protozoa. It is commonly used as a primary treatment for diarrhea in dogs [13], and has been used to treat foals with hemorrhagic enteritis caused by *Clostridium difficile* [14]. Metronidazole is also prescribed to *Clostridial* diarrhea in adult horses. Although historically metronidazole has been associated with a low incidence of diarrhea when used in adult horses [15], its effect on the bacterial communities of the GIT has not yet been reported. While culture-based methods have traditionally been used to assess the effects of antibiotics, the use of Next Generation Sequencing and metabolomics can potentially provide greater insight regarding the changes in the microbial populations and their functional effect on the metabolism of the horse’s hindgut [3, 16, 17]. The objective of this study was to characterize the changes in the cecal and fecal microbiome and metabolome of the horse before and after metronidazole administration.

## Materials and methods

This study was approved by Texas A&M University Institutional Animal Care and Use Committee (IACUC; Protocol number 2014–0123).

### Study population and sample collection

Five horses belonging to the Department of Animal Science at Texas A&M University with indwelling cecal cannulas were used as subjects. The horses had been cannulated approximately 10 years prior to this study, and had received no antibiotics or other medications (excluding routine vaccinations and anthelminthics) for the previous 12 months. The horses were housed on a dry lot, fed free choice coastal hay and a commercial pelleted concentrate (Nutrena Safe Choice, Cargill, Minnetonka, MN) at 0.5% of body weight for the duration of the study. Age, breed, sex, and weight of the study participants is summarized in Table 1.

**Table 1:** Age, breed, sex and weight of study participants.

Horse	Age (years)	Breed	Sex	Weight (kg)
1	19	Quarter Horse	Gelding	500
2	12	Mixed	Gelding	512
3	24	Quarter Horse	Gelding	413
4	13	Quarter Horse	Gelding	602
5	15	Mixed	Gelding	654

Fecal samples were collected immediately after natural elimination. Cecal samples were collected by removing the plug of the cannula while horses were restrained in stocks and siphoning 5 mls of cecal ingesta, including both the particulate and liquid fractions. Samples were placed on ice for transport to the laboratory, where they were refrigerated at 4°C prior to DNA extraction. Samples were collected before (study days referred to as Dminus52, Dm28, Dm14, and 0) and after metronidazole administration (referred to as D7, D14, D28, and D52). Metronidazole (Unichem Pharmaceuticals, Hasbrouck Heights, NJ) was administered after sample collection on Day 0 at a dose of 15 mg/kg PO BID for 3 days instead of 7 days as planned in the study protocol due to development of complications (see below in result section for details). This resulted in an additional sampling point for feces on Day 3.

### DNA extraction

One hundred mg of feces or cecal contents (particulate and liquid fractions) was aliquoted into a sterile 1.7 ml tube (Microtube, Sarstedt AG & Co, Numbrecht, Germany) containing 150 μl of 0.1 mm zirconia-silica beads and 100 μl of 0.5 mm zirconia-silica beads (BioSpec Products Inc., Bartlesville, OK, USA). Samples were then homogenized (FastPrep-24, MP Biomedicals, Irvine, CA, USA) for a duration of 1 minute at a speed of 4 m/s. DNA was extracted using the PowerSoil DNA Isolation Kit (MO BIO, Carlsbad, CA, USA) following the manufacturer’s instructions.

### Sequencing of 16S rRNA genes

Sequencing of the V4 region of the 16S rRNA gene was performed at MR DNA (www.mrdnalab.com, Shallowater, TX, USA) on an Illumina MiSeq platform (Illumina Inc., San Diego, CA) following the manufacturer’s guidelines using 515F (5’-GTGCCAGCMGCCGCGGTAA-3’) and 806R (5’- GGACTACVSGGGTATCTAAT-3’). Briefly, the PCR reaction was performed in a single-step 30 cycle PCR using the HotStarTaq Plus Master Mix Kit (Qiagen, USA) under the following conditions: 94°C for 3 minutes, followed by 28 cycles (5 cycles used on PCR products) of 94°C for 30 seconds, 53°C for 40 seconds and 72°C for 1 minute, after which a final elongation step at 72°C for 5 minutes was performed. Using Illumina TruSeq DNA’s protocol, a DNA library was set up and Illumina MiSeq was used for sequencing according the manufacturer’s guidelines.

### Analysis of sequences

QIIME v1.9 (Quantitative Insights into Microbial Ecology) was used for analysis of the sequences. After sequencing, barcodes and primers were removed and short (<150 bp), ambiguous, homopolymeric sequences were depleted from the dataset. USEARCH was used to identify and remove chimeric sequences. Operational taxonomic units (OTUs) were assigned based on at least 97% sequence similarity to the Greengenes database (v13.5) using an open reference approach. Sequences determined to be mitochondria, chloroplasts, unassigned, or those belonging to the phylum cyanobacteria were excluded from further analysis.

### Enteric pathogen testing

Both cecal and fecal samples were tested for the presence of *Salmonella* and *Clostridial* toxins. For *Salmonella* PCR, a tetrathionate green broth enrichment method was used prior to extraction, and qPCR performed as previously described [18]. Commercially available ELISAs for *Clostridium perfringens* enterotoxin and *Clostridium difficile* toxins A and B were performed according to the manufacturer’s instructions (TechLab, Blacksburg, VA, USA).

### Metabolomics analysis

Samples were stored at −80°C until shipped on dry ice to the West Coast Metabolomics Core (University of California, Davis, CA, USA) for untargeted analysis [19]. Samples were lyophilized for 24 hours and weighed in a 1.5 ml Eppendorf tube. One ml of a 3:3:2 extraction mixture of degassed acetonitrile (Fisher, Ottawa, Canada, A9554), isopropanol (Fisher, Ottawa, Canada, A461212) and water (Fisher, Ottawa, Canada, 7732-18-5) was used to re-suspend 4 mg of lyophilized sample. Three millimeter grinding beads (Next Advance, Troy, NY, USA) were added to disrupt the sample at 1500 rpm for 30 seconds followed by shaking at 4°C for 5 minutes and centrifugation for 2 minutes at 14,000 rcf. The supernatant was separated into two aliquots of 475 ul for dry down in a Centrivap cold trap vacuum overnight at room temperature (Labconco, Kansas City, MO, USA). Dried supernatant was resuspended in 500 ul of 50% aqueous acetonitrile (Fisher, Ottawa, Canada, A9554) and centrifuged at 14,000 rpm for 2 minutes. The supernatant was dried down overnight in a Centrivap overnight at room temperature. A volume of 10 ul of 40 mg/ml solution of methoxyamine hydrochloride (Sigma-Aldrich, St. Louis, MO, USA, 89803) in pyridine (99.99%) (Sigma-Aldrich, St. Louis, MO, USA 270970) was added to the samples and shaken for 90 minutes at 30°C. Next, 90 uL of N-methyl-N-trimethylsilyltrifluoroacetamide (MSTFA) with 1% trimethylchlorosilane (TMCS) (Sigma-Aldrich, St. Louis, MO, USA 69479) and Fatty Acid Methyl Esters (FAMEs) retention indexing markers (see next paragraph) were added with a ratio 100:0.001 was added to each sample and shaken for 37°C for 30 minutes for trimethylsilylation of acidic protons. The reaction mixture was placed in a 2 ml clear glass auto-sampler vial with micro-insert (Agilent, Santa Clara, CA, USA, 5185–5946) and closed with a 11mm T/S/T crimp cap (Thomas Scientific, Swedesboro, NJ, USA, 11-0038A).

Quality control was assessed by retention indexing with Fatty Acid Methyl Esters (FAMEs) and the use of blanks and quality control samples. A mixture of internal retention index markers using 13 FAME markers dissolved in chloroform (0.8mg/ml C8-C16 or 0.4 mg/ml C18-C30) (Acros, Morris Plains, New Jersey, USA, AC4235500100) was added to each sample (S1 File) as listed above during derivatization. During data acquisition, both blank samples and a quality control mix were injected every 10 samples. Blank samples were used to check for carry over and also in data processing for blank subtraction. The quality control mix included 28 compounds used in 6 concentrations (S1 File) that were dried down and derivatized as previously described.[19]

An Agilent 6890 GC equipped with a Gerstel automatic liner exchange system (ALEX) that includes a multipurpose sample (MPS2) dual rail, and a Gerstel CIS cold injection system (Gerstel, Muehlheim, Germany) with temperature program was used as follows: 50°C to 275°C final temperature at a rate of 12°C/s and hold for 3 minutes. Injection volume is 0.5 μl with 10 μl/s injection speed on a splitless injector with purge time of 25 seconds. Liner (Gerstel #011711-010-00) is changed after every 10 samples, (using the Maestro1 Gerstel software vs. 1.1.4.18). Before and after each injection, the 10 μl injection syringe is washed three times with 10 μl ethyl acetate. For gas chromatography, a 30 m long, 0.25 mm i.d. Rtx-5Sil MS column (0.25 μm 95% dimethyl 5% diphenyl polysiloxane film) with additional 10 m integrated guard column was used (Restek, Bellefonte, PA, USA). 99.9999% pure Helium with built-in purifier (Airgas, Radnor Pennsylvania, USA) was set at constant flow of 1 ml/min. The oven temperature was held constant at 50°C for 1 min and then ramped at 20°C/min to 330°C for 5 minutes. A Leco Pegasus IV time of flight mass spectrometer controlled by the Leco ChromaTOF software vs. 2.32 (St. Joseph, MI, USA) was used for mass spectrometry. The transfer line temperature between gas chromatograph and mass spectrometer was set to 280°C. Electron impact ionization at 70V was employed with an ion source temperature of 250°C. Acquisition rate was 17 spectra/second, with a scan mass range of 85–500 Da.

Raw data files were processed using ChromaTOF v.2.32. BinBase algorithm matched spectra to database compounds, and quantification was reported by peak height of an ion at the specific retention index characteristic of the compound across all samples. Peak heights were normalized by average total peak-sums for detected compounds across each sample group. Metabolites were cross referenced using their compound number to the KEGG database (https://www.genome.jp/kegg/pathway.html) in order to identify their metabolic pathways such as metabolism, cellular processes, and others. Metabolomic data has been submitted to metabolomicsworkbench.org under the submission ST001248.

### Statistical analysis

#### Microbiome analysis

Prior to analysis, data were tested for normality using the Shapiro-Wilk test (JMP Pro 14, SAS, Marlow, Buckinghamshire). As data followed a non-normal distribution, non-parametric measures were used throughout the study. Adjustments for multiple comparisons were made with either a Dunn’s post-test or Fischer’s least significant differences. P- and q-values <0.05 were considered statistically significant.

Alpha diversity was calculated using observed OTUs, Shannon, and Chao1 metrics to compare species richness and evenness. Statistical analysis of alpha diversity indices was performed using the software package PRISM (PRISM 7, GraphPad Software Inc., San Diego, CA). A Friedman’s test followed by a Dunn’s multiple comparison post-test were performed to assess differences in alpha diversity metrics between study days.

Beta diversity (bacterial community composition) was calculated using both weighted and unweighted UniFrac metrics to measure similarity between samples, and visualized for clustering with Principle Coordinate Analysis (PCoA) plots. An Analysis of Similarity test (ANOSIM) within the PRIMER 6 (PRIMER-E Ltd. Luton, UK) software package was performed on the beta diversity distance matrices to assess the significance of the differences in the bacterial community composition.

The abundance of bacterial taxa in the cecal and fecal samples was evaluated using a Friedman’s test (PRISM 7, GraphPad Software Inc., San Diego, CA) followed by a Dunn’s multiple comparison post-test. Only bacterial taxa present in at least 50% of the samples of at least one time point were included in the analysis. P values of <0.05 and q values of <0.1 were considered significant.

Linear discriminant analysis effect size (LEfSe) using the web-based program Calypso v8.62 (http://cgenome.net/wiki/index.php/Calypso) was performed to analyze the abundance of bacterial taxa and their associations with study day. A cut-off threshold of 3.5 was set for significance.

#### Metabolomics analysis

MetaboAnalyst 4.0 (Xia Lab, McGill University, Canada) was used to analyze metabolomics data. The peak intensity data table contained peak heights normalized against the average total peak sums. Data was not filtered, normalized or transformed, but was subjected to Pareto scaling. Principle Component Analysis (PCA) plots were used to display metabolic composition of individual horses and a heat map was used to display hierarchical clustering of the significant metabolites. An ANOVA test followed by a Fisher’s least significant differences was used to determine, which time points in the cecal or fecal samples differed significantly over study days Dm28-D28.

## Results

### Study population and sample collection

Metronidazole administration began as planned on Day 0 after sample collection. By the evening treatment time on Day 3, all horses had become inappetent and had developed significant skin scalding associated with the canula site. Metronidazole was discontinued after a total of 5 doses had been administered (Day 3 of administration) due to the investigators’ concern of impending colitis. An extra fecal sample was collected on Day 3, while cecal samples were not obtained at this time point due to the horses’ level of discomfort with canula manipulation. The skin scalding was treated with local wound care including gentle washing of the skin and application of an emollient ointment around the canula site. All horses were normal on physical examination and their appetites had recovered within 48 hours of discontinuing metronidazole administration. Skin scalding resolved by Day 7. Horse 3 developed mild signs of colic (i.e., flank watching, pawing) on Day 13 and was moved from the research facility to the hospital for further diagnostics and monitoring. The horse’s vital parameters (i.e., temperature, heart rate, respiratory rate, mucous membrane color) remained within normal limits and it was producing normal feces. Palpation per rectum, passage of a nasogastric tube and preliminary bloodwork to assess hydration status (i.e., packed cell volume, total protein and lactate) were performed. No abnormalities were detected. Due to the mild nature of the colic, Horse 3 received 7 L of oral fluids with electrolyte supplementation and one dose of flunixin meglumine (1.1 mg/kg iv). The horse remained hospitalized for 24 hours during which time his vital parameters remained within normal limits, he was passing normal manure and the displayed no symptoms of colic. The investigator (CA) elected to return the horse to the research facility in the afternoon on Day 14. After scheduled sample collection, the horse was found deceased 4 hours later. The Institutional Animal Care and Use Committee was notified, and a necropsy was performed. The cause of death was determined to be due to typhylocolitis. Due to the death of this subject, data from Horse 3 is missing from the Day 28 and 52 time point data sets. The remaining 4 horses completed the study with no other adverse events. Due to these events, baseline samples include Days m52, m28, m14 and 0. Day3 represents the period when horses were treated with metronidazole and Days 7, 14, 28 and 52 represent the time period following discontinuation of metronidazole.

### Sequence analysis

The total sequence analysis yielded 6,288,464 quality sequences for all analyzed samples (n = 81, mean ± SD = 77,635 ± 15, 531). For cecal content samples (n = 38), the mean quality sequences and standard deviation was 77,054 ± 18841. For fecal samples (n = 43), the mean quality sequences and standard deviation was 78,212 ± 12202. Samples were rarefied to an even depth of 40,005 reads per sample. The sequences were deposited in the National Center for Biotechnology Information (NCBI) Sequence Read Archive (SRA) under the accession number SRP119693.

### Beta diversity

**Fig 1:**
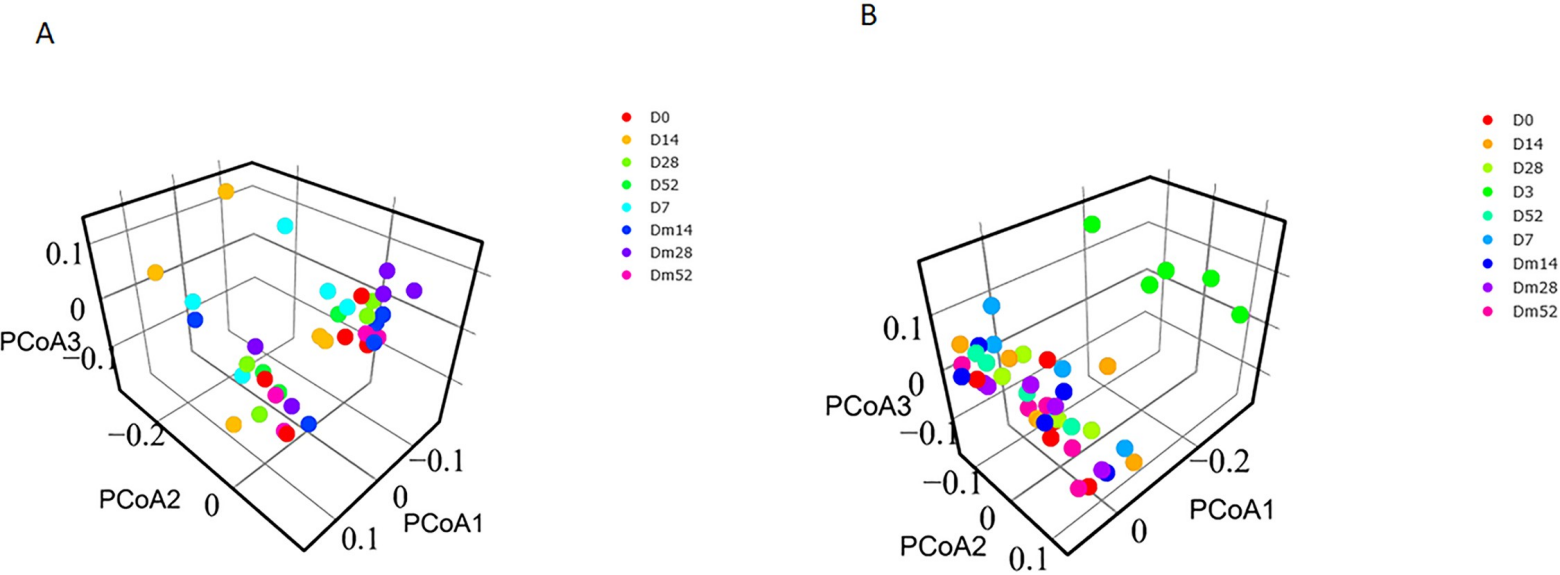
Beta diversity: Principal coordinates analysis (PCoA) plot based on unweighted Unifrac distances. **(A)** Cecal samples. **(B)** Fecal samples.

Beta diversity (Fig 1A), as measured by Unifrac distances showed no significant differences for cecal samples (unweighted, R = 0.072, p = 0.108; weighted, R = 0.159, p = 0.001). Fecal samples (Fig 1B) showed visible clustering on Day 3 (ANOSIM, R = 0.152, p = 0.004), which was confirmed by pairwise comparisons. Beta diversity on Day 3 was significantly different from all other time points (Dm52, Dm28, Dm14, D0, D7, D14, D28, and D52) on both weighted and unweighted Unifrac distances in the fecal samples (Table 2).

**Table 2:** The results of pairwise ANOSIM testing of unweighted and weighted Unifrac distances for fecal samples between Day 3 and all other study days.

Unifrac distances	Unweighted		Weighted	
Study Days	R statistic	P value	R statistic	P value
D3, Dm52	0.92	0.008	0.744	0.008
D3, Dm28	0.892	0.008	0.752	0.008
D3, Dm14	0.896	0.008	0.716	0.008
D3, D0	0.884	0.008	0.764	0.008
D3, D7	0.868	0.008	0.716	0.008
D3, D14	0.64	0.008	0.572	0.032
D3, D28	0.838	0.008	0.575	0.024
D3, D52	0.913	0.008	0.638	0.024

### Alpha diversity

**Fig 2:**
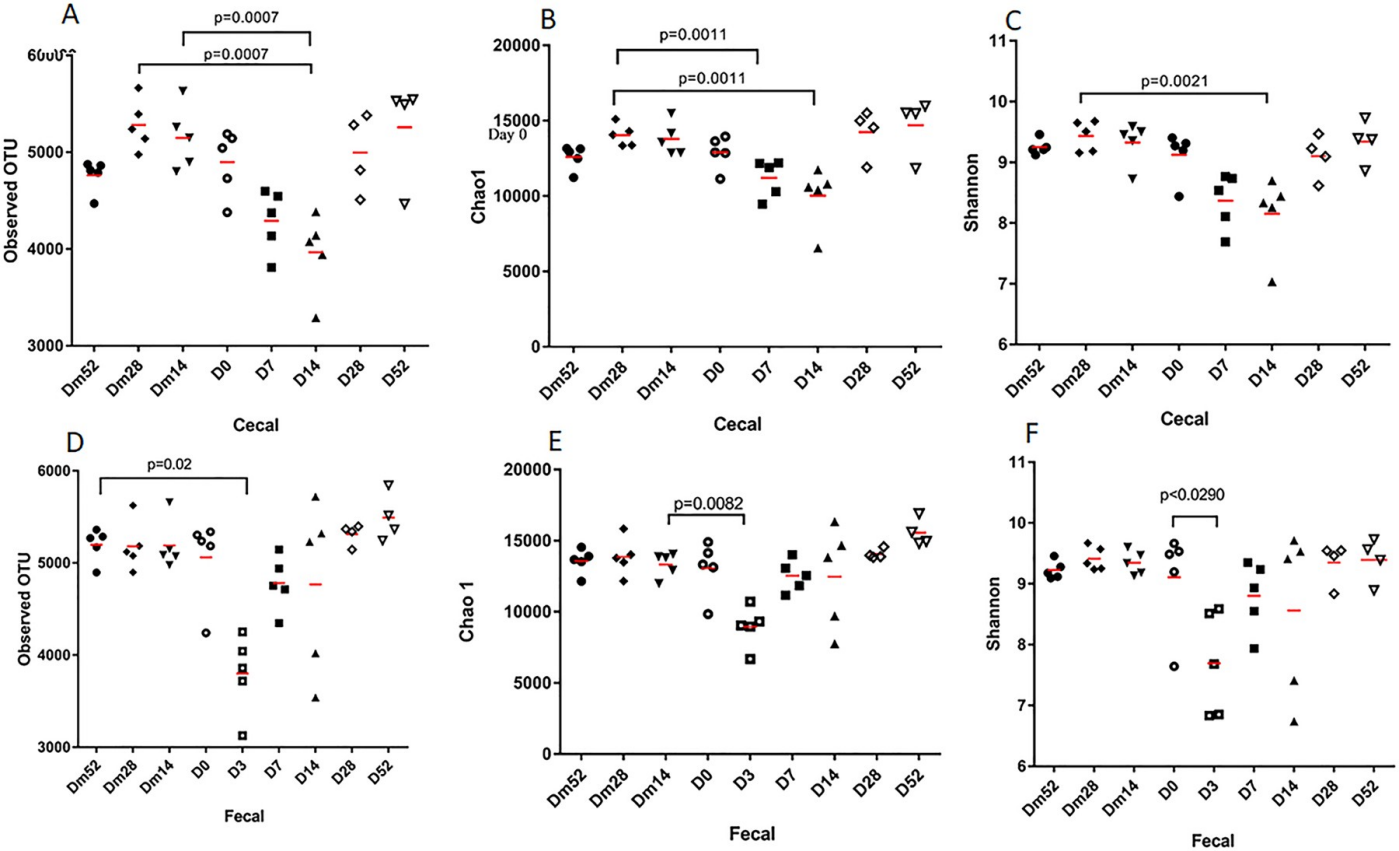
Alpha diversity measures by study day. **(A)** Observed OTUs of cecal samples. **(B)** Chao1 of cecal samples. **(C)** Shannon of cecal samples. **(D)** Observed OTUs of fecal samples. **(E)** Chao1 of fecal samples. **(F)** Shannon of fecal samples.

Alpha diversity indices (observed OTUs, Chao1, and Shannon) for cecal and fecal samples are reported in Fig 2A–2F. Due to the death of Horse 3 on Day 14, analysis by Friedman’s test on Days 28 and 52 could not be completed, but the data for the remaining 4 horses is provided. For cecal samples, the following significant differences were noted: Observed OTUs between Dm28 and D14 (p = 0.0007), Dm14 and D14 (p = 0.0007); Chao 1 between Dm28 and D14 (p = 0.001) and Dm28 and D7 (p = 0.001); and Shannon for Dm28 and D14 (p = 0.0021). For fecal samples, the following differences were noted: observed OTUs Dm52 and D3 (p = 0.015), Chao 1 between Dm14 and D3 (p = 0.008), and Shannon between D0 and D3 (p = 0.0209).

### Univariate analysis

**Fig 3:**
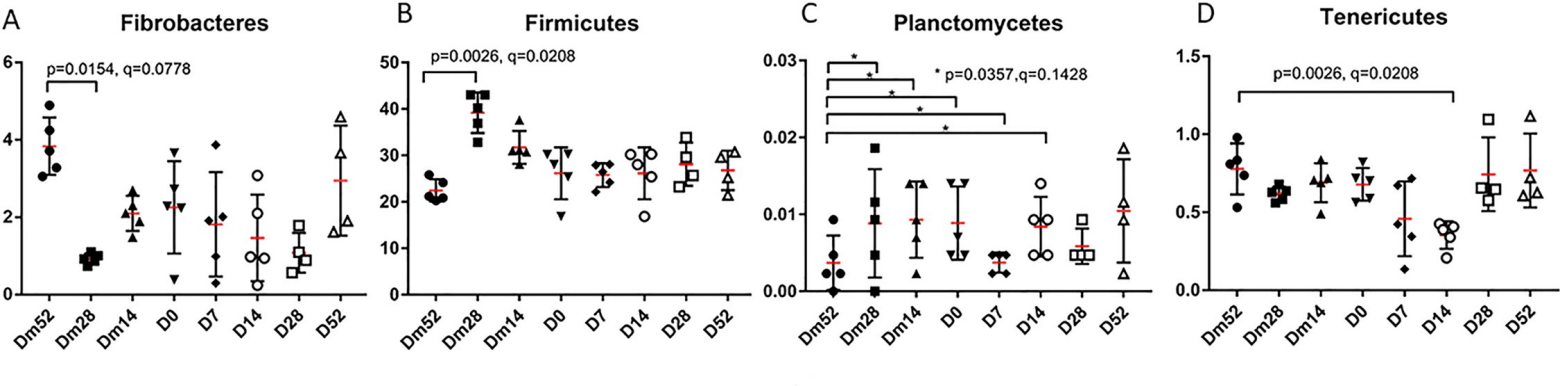
The median abundance (%) of significantly different cecal phyla across study days. **(A)** Fibrobacteres. **(B)** Firmicutes. **(C)** Planctomycetes. **(D)** Tenericutes.

In the cecal samples, only the phyla Firmicutes (increased in abundance from Dm52 to Dm28; p = 0026, q = 0.0208) and Tenericutes (decreased in abundance from Dm52 to D14; p = 0026, q = 0.0208) were significantly different between study days after adjustments for multiple comparisons had been made. Other phyla such as Fibrobacteres (p = 0.0154, q = 0.0778) and Planctomycetes (p = 0.0357, q = 0.1428) had significantly different p values, but did not achieve significance after adjustments for multiple comparisons were made (Fig 3A–3D). At the family level, there were 27 taxa that were significantly different on a Friedman’s test, but not after adjustment for multiple comparisons. Complete information regarding cecal content taxa at the phylum and family levels are included in S1 and S2 Tables.

**Fig 4:**
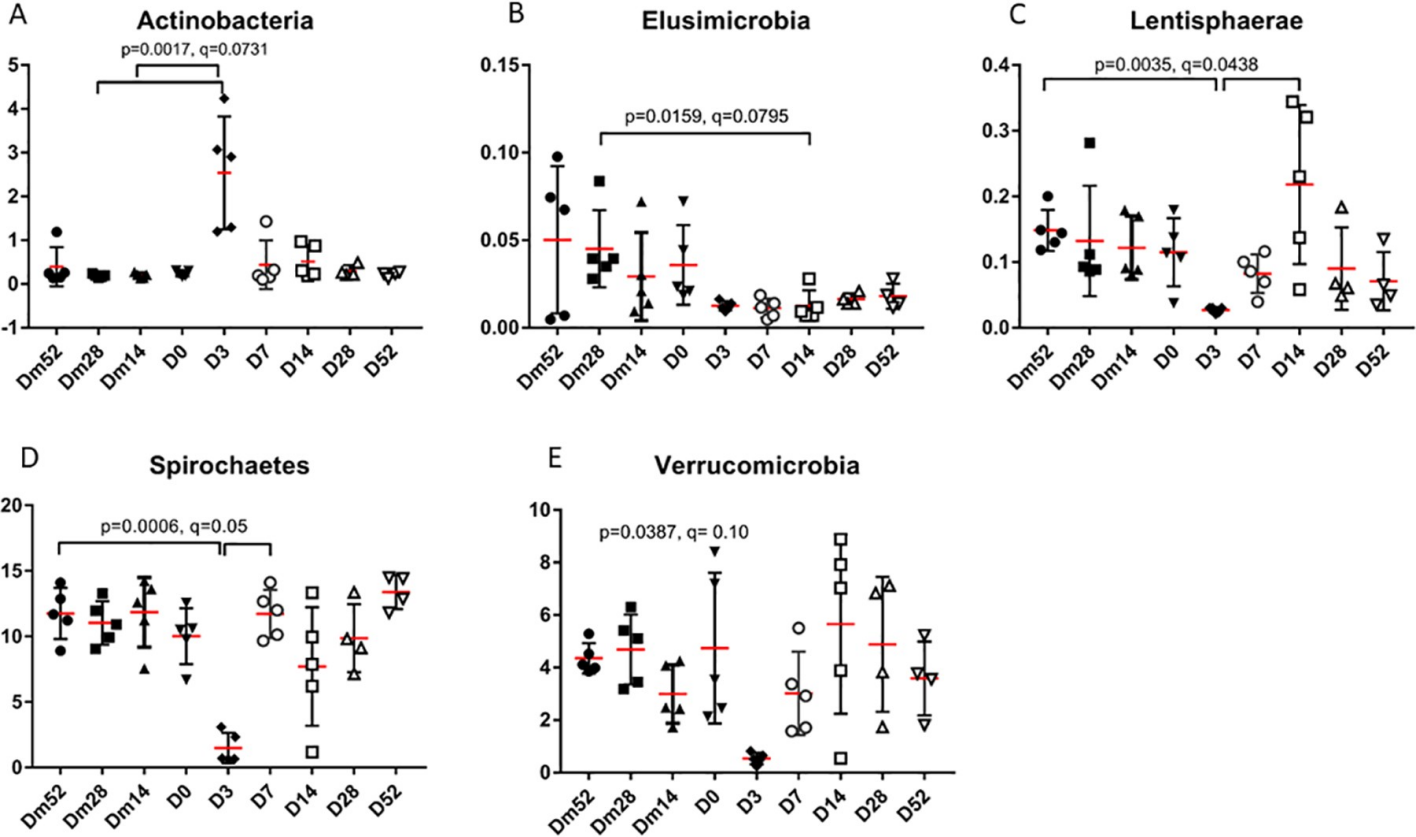
The median abundance (%) of significantly different fecal phyla across study days. **(A)** Actinobacteria. **(B)** Elusimicrobia. **(C)** Lentisphearae. **(D)** Spirochaetes. **(E)** Verrucomicrobmia.

In fecal samples, Lentisphaerae (p = 0.0035, q = 0.0438) and Spirochaetes (p = 0.0060, q = 0.05) were decreased on D3. Elusimicrobia (p = 0.0159, q = 0.0795) was significantly decreased on D14 compared to Dm28, but this difference was not evident once adjusted for multiple comparisons. Actinobacteria (p = 0.0117, q = 0.0731), Fibrobacteres (p = 0.0440, q = 0.1000), SR1 (p = 0.0212, q = 0.0850), Synergistetes (p = 0.0266, q = 0.0850), and Verrucomicrobia (p = 0.0387, q = 0.1000) were only significantly different on the Friedman’s but not the Dunn’s post-test (Fig 4A–4E). Complete information regarding fecal taxa at the phylum and family level is included in S3 and S4 Tables.

### Linear discriminant analysis effect size

Linear discriminant analysis effect size (LEfSe) was used to elucidate taxa associated with study day. A linear discriminant analysis (LDA) score > 3.5 was considered significant. In the cecal content samples, only 2 phyla (i.e., Firmicutes and Fibrobacteres) met this threshold. At the family level in cecal samples, 5 taxa (i.e., Fibrobacteriaceae, Unclassified Y2, Ruminococcaceae, Unclassified Clostridiales, and Lachnospiraceae) were more abundantly expressed prior to metronidazole administration, whereas 3 taxa (i.e., Porphyromonadaceae, Veillonellaceae, and Succinivibrionaceae) were more abundantly expressed after metronidazole administration. Table 3 contains complete information regarding the taxa, study day, and LDA scores from cecal content samples.

**Table 3:** Linear discriminant analysis of bacterial taxa in cecal samples and their associations with study day. Only LDA scores of >3.5 are shown.

Taxa	LDA	Time point
**Phylum**		
Firmicutes	4.92	Dm28
Fibrobacteres	4.16	Dm52
**Family**		
Fibrobacteraceae	4.15	Dm52
Unclassified_YS2	3.6	Dm52
Ruminococcaceae	4.37	Dm28
Unclassified_Clostridiales	4.39	Dm28
Lachnospiraceae	4.57	Dm28
Porphyromonadaceae	3.52	D14
Veillonellaceae	4.32	D14
Succinivibrionaceae	4.4	D14

In fecal samples, 2 taxa at the phylum level (i.e., Elusimicrobia and Euryarchea) and 3 taxa at the family level (i.e., Clostridicaceae, Methanocorpusculaceae, and Ruminococcaceae) were associated with an LDA score of greater than 3.5 at baseline. Fecal taxa at the phylum level significantly associated study days following metronidazole administration included: D3: Actinobacteria and Protobacteria; D14: Verrucomicrobia and Lentisphaerae; and D28: Planctommycetes and Synergistetes. At the family level, Alcaligenaceae, Corynebacteriaceae, Neisseriaceae, Actinomycetaceae, Porphyomonadaceae, Tisserellaceae, Enterobacteriaceae, Pastuerellaceae, Streptococcaceae, Aerococcaceae, Lacobacillaeceae, and Aeromondaceae were more abundant on D3, whereas Methanobacteriaceae and RFP12 were more abundant on D14. Bacillaceae and Planococcaceae were associated with D28. Table 4 displays the results of LEfSe analysis for fecal samples at both the phylum and family levels.

**Table 4:** Linear discriminant analysis of bacterial taxa in fecal samples and their associations with study day. Only LDA scores of >3.5 are shown.

Taxa	LDA	Time point
**Phylum**		
Elusimicrobia	3.92	Dm52
Euryarchaeota	4.12	Dm28
Actinobacteria	4.10	D3
Proteobacteria	5.06	D3
Verrucomicrobia	4.43	D14
Lentisphaerae	3.53	D14
Planctomycetes	3.72	D28
Synergistetes	3.66	D28
**Family**		
Clostridiaceae	3.76	Dm52
Methanocorpusculaceae	4.04	Dm28
Ruminococcaceae	4.37	Dm28
Alcaligenaceae	3.61	D3
Corynebacteriaceae	3.62	D3
Neisseriaceae	3.64	D3
Actinomycetaceae	3.90	D3
Porphyromonadaceae	3.93	D3
Tissierellaceae	4.00	D3
Enterobacteriaceae	4.12	D3
Pasteurellaceae	4.18	D3
Streptococcaceae	4.34	D3
Aerococcaceae	4.35	D3
Lactobacillaceae	4.60	D3
Aeromonadaceae	4.68	D3
Methanobacteriaceae	3.77	D14
RFP12	4.45	D14
Bacillaceae	4.01	D28
Planococcaceae	4.47	D28

### Testing for enteric pathogens

All horses were negative for *Salmonella* PCR in fecal samples across all time points. Four horses (i.e., horses 1, 2, 4, and 5) were positive for *Salmonella* by PCR testing in cecal content samples on Day 7. Horses 1, 2, and 4 remained positive on Day 14, but tested negative on Day 28. Horse 5 remained positive at D52, the final time point of the study. Horse 3 was positive on Day 14, and subsequently died later that day. Serotyping revealed *Salmonella enterica* serotype Newport in one horse (horse 1), *Salmonella enterica* serotype anatum in 3 horses (horses 2, 3, and 4) and multiple serotypes in one horse (horse 4). All horses were negative for *Clostridium perfringens* and C. *difficile* toxins in both cecal and fecal samples at all time points.

### Metabolomic analysis of cecal and fecal samples

Using an untargeted approach, a total of 554 unique metabolites weredetected, of which 223 were named. Metabolomic data has been submitted to metabolomicsworkbench.org under the submission ST001248.

**Fig 5:**
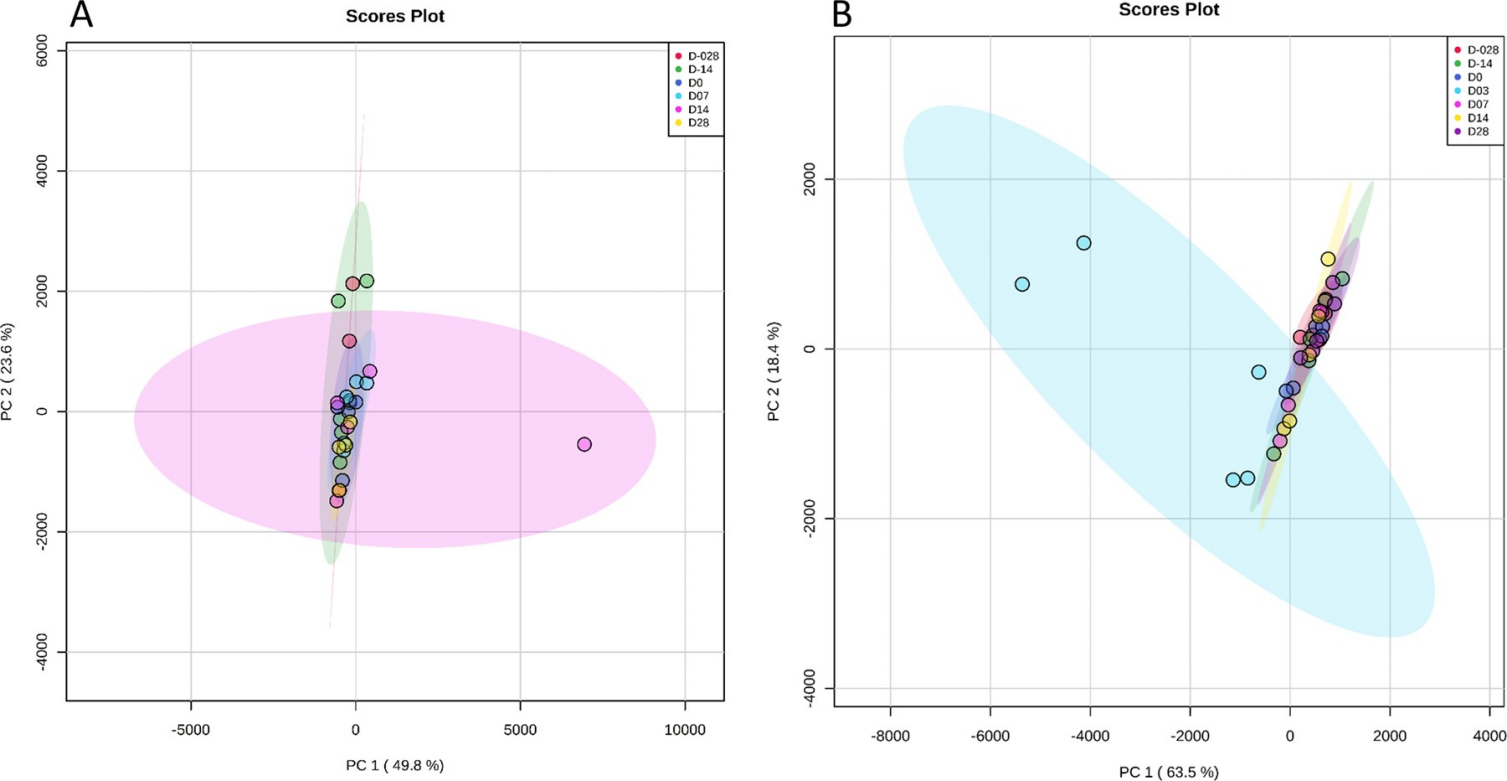
Principal component analysis of the cecal and fecal metabolites by study day. The shaded ellipses represent the 95% confidence interval of the metabolite profile. **(A**) Cecal metabolites. **(B)** Fecal metabolites.

Only named metabolites were included in the analysis. Metabolites were examined in the cecum on Days m28, m14, 0, 7, 14 and 28. Metabolites were examined in the feces on Days m28, m14, 0, 3, 7, 14 and 28. Samples were not collected on Day m52 and 52 for metabolomic analysis, and on Day m28 Horse 5 did not have enough sample for analysis. Metabolites were analyzed using PCA score plots and heat maps. In the cecal samples, the PCA plot of all named metabolites (Fig 5A) indicated that samples on D14 were different from other study days. This is due to the presence of an outlier, Horse 3, whose metabolite profiles were presumably altered by the presence of gastrointestinal disease and subsequent death. No cecal metabolites were significantly different by study day after adjustments for multiple comparisons were made.

**Fig 6:**
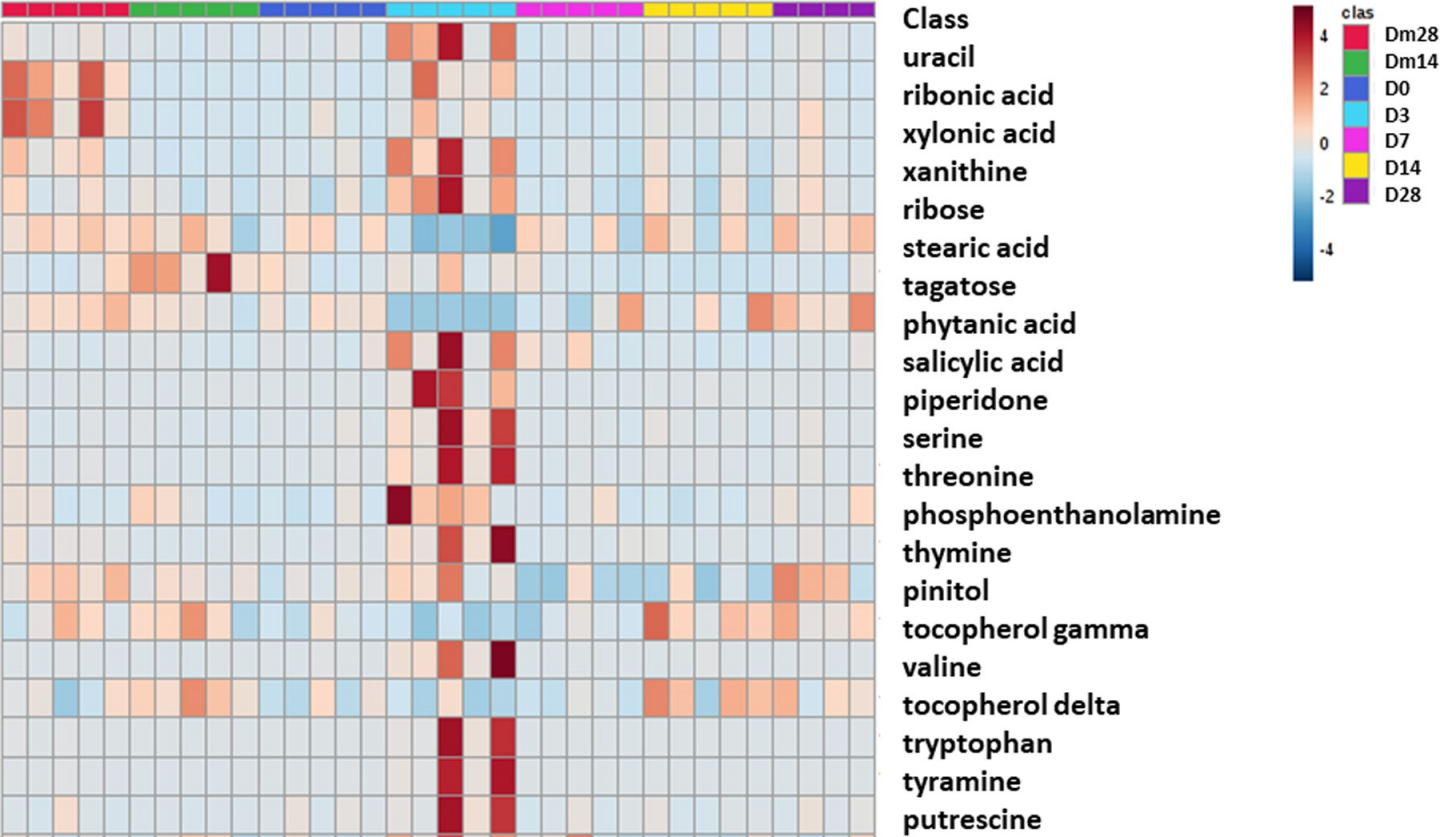
Heatmap of the significant fecal metabolites from Days m28, m14, 0, 3, 7, 14, 28. Each column represents a sample, sorted by study day, and each row represents one metabolite. Color intensity indicates intensity the increase (red) or the decrease (blue) in metabolite concentration.

In fecal samples, Day 3 samples clustered distinctly from all other study days in the PCA scores plot (Fig 5B). Mulitvariate analysis of all the named metabolites found 21 fecal metabolites that were significantly different across study days following adjustment for multiple comparisons. A heatmap of the significantly different named fecal metabolites (Fig 6) indicated a visible change in concentration relative to study day. Metabolites related to nucleic acid metabolism (thymine, uracil, xanthine), amino acid metabolism (putrescine, salicylic acid, serine, threonine, tryptophan, tyramine, valine), lipid metabolism (phosphoenolamine, ribonic acid) and carbohydrate metabolism (ribose) all had significantly elevated levels on Day 3. Other metabolites related to lipid metabolism (phytanic acid, stearic acid) and the metabolism of cofactors and vitamins (δ-tocopherol, γ-tocopherol) were decreased on Day 3 in relation to other study days. Information regarding the significant fecal metabolites and their respective KEGG pathways is provided in S5 Table. Information regarding the changes in significant fecal metabolites by study day is provided in S1–S5 Fig.

## Discussion

This study utilized a herd of horses with cecal cannulas to investigate the effects of metronidazole on the equine cecal content and fecal microbiome and metabolome. The horses had been cannulated approximately 10 years earlier, and had experienced no disruption in environment, feed, housing, or exposure to new horses for 6 months prior to and during the study period. While the use of cannulated animals is commonplace in nutritional studies, it is somewhat novel in microbiome and metabolome studies. The presence of a cannula in the cecum does allow ingresss of oxygen into the cecum during sampling periods. However, in other species it has been demonstrated that anaerobic conditions are quickly restored once the plug is replaced [20]. In ruminant species such as goats and cattle, the presence of the cannula has not significantly altered diversity indices or taxonomy of major phyla [21–23]. Because horses were sampled every 7–14 days, the short-term introduction of oxygen likely had a minimal effect. Although unlikely to significantly alter the results, the use of cannulated animals in this study should be interpreted accordingly.

The horses experienced adverse effects at two time points in the study, Days 3 and 14. The first adverse event occurred on Day 3 in all 5 horses and was manifested as inappetance and skin scalding at the canula site. Inappetance can be an early manifestation of gastrointestinal disease, and the skin scalding was presumably due to a change in the pH of the cecal fluid. Previously studies using these same 5 cannulated horses for grain overload studies indicated that decreasing the pH of the cecal fluid could result in fluid leaking from the canula and subsequent skin scalding [24]. Out of caution, the authors elected to suspend metronidazole administration after only 5 of the 14 doses had been given. All the horses returned to an apparently healthy state within 48 hours, and none of the horses showed symptoms of gastrointestinal disease until later in the study. The second adverse event occurred on Day 13 when Horse 3 experienced an episode of colic and died 24 hours later. Post-mortem examination indicated colitis as the cause of death. As the study had been suspended 11 days earlier and the remaining horses appeared healthy, the investigators chose to continue sampling from the remaining horses and use the data from the deceased horse. Due to the loss of Horse 3, samples from Days 28 and 52 only included horses 1, 2, 4, and 5. Friedman’s analysis for these two time points was not possible, although the data from these 4 horses is presented.

Similarly to reports in dogs, metronidazole decreased the diversity of the microbiome in the horses evaluated, but at different time points for cecal content and fecal samples [13, 25]. The OTU, Shannon, and Chao 1 metrics were consistently reduced in cecal samples on Day 14, whereas fecal samples had the lowest alpha diversity measures on Day 3. Recovery of alpha diversity indices occured by Day 28 in both sample types. Beta diversity was unaffected by metronidazole administration in the cecal samples. However fecal samples were distincly clustered on Day 3 compared to the other time points in the weighted and unweighted PCoA plots. Metronidazole had the greatest effects on alpha and beta diversity on Days 3 and 14, which is consistent with the clinical appearance of gastrointestinal disease in the study subjects.

At baseline, both cecal and fecal samples appeared to have a similar microbial community composition. Bacteroidetes and Firmicutes, important phyla for fiber degradation and affected by the presence of gastrointestinal disease in other studies [2, 3, 5, 17], constituted the majority of the phyla. Spirochaetes, Fibrobacteres, Proteobacteria, Tenericutes, and Verrucomicrobia comprised approximately 1% or more of the total bacteria. The cecum had a greater percentage of Bacteroidetes, while the feces contained more Fibrobacteres and Spirochaetes. This is consistent with other reports comparing the anatomic compartments of the GIT in normal horses [26, 27] and also in previous reports of cannulated horses [28].

Over time, the bacterial community composition was altered by metronidazole. In this study, the abundance of Bacteroidetes remained unchanged in either sample type. In the cecum, the phyla Firmicutes, Fibrobacteres, Planctomycetes, and Tenericutes were affected. There was a significant change in the abundance of these phyla from Dm52 to Dm28, which is likely related to normal variation in the microbiota over time, ambient temperature, and season [29–31]. The magnitude of this change likely overshadowed the downward trend in abundance of Fibrobacteres, Firmicutes, Planctomycetes, and Tenericutes on Days 7 and 14 due to metronidazole administration. The alterations in the Firmicutes occurred within the class Clostridia and the families Other, Unknown, Christensenellaceae, Dehalobacteriaceae, Lachnospiraceae, Ruminococcaceae, and Veillonellaceae. A similar effect was noted with the phylum Fibrobacteres and the family Fibrobacteraceae prior to metronidazole. These changes did not achieve significance after adjustments for multiple comparisons were made at the family level. This finding was reinforced by the LEfSe analysis. These changes may have reached significance if the number of horses used in the study would have been greater or if metronidazole administration had not been terminated early on Day 3.

In the feces, the phyla Actinobacteria, Elusimicrobia, Lentispherae, Spirochaetes, and Verrucomicrobia were affected by metronidazole administration. All of these phyla, except Actinobacteria, decreased in abundance on either Days 3, 7, or 14. The abundance of Actinobacteria was significantly elevated on Day 3 in the feces compared to other study days, with the family Bifidobacteriaceae accounting for majority of this increase. Actinobacteria is a gram-positive facultative anaerobe organism that accounts for a relatively small percentage of the total number of bacteria. However, a similar increase was reported after metronidazole use in dogs and rats [13, 32]. The significance of the decrease in the abundance Elusimicrobia, Lentisphereae, and Spirochaetes at various time points after metronidazole administration is unknown. Lentisphereae and Spirochaetes are anaerobes or facultative anaerobes, which were likely affected by metronidazole. Lentispherae is commonly found in the gut of mammals but comprises less than 1% of all taxa. Spirochaetes, however, represent 6.5% and 11.3% of the cecal content and fecal samples, respectively. Metronidazole caused a significant decrease in the Spirochaetes at both the phylum and family level on D3 but quickly recovered by the last sampling points. The functional role of these three phyla and their response to metronidazole are yet to be fully elucidated.

Univariate analysis and LEfSe indicated that Actinobacteria and Verrucomicrobia were affected on Day 3 in the feces. Verrucomicrobia showed a marked decline in both cecal content and fecal samples on Days 3 and 7, although this result did not reach significance with the Dunn’s post-test (q = 0.0717). Verrucomicrobia is a phylum of strict anaerobes that maintains the mucus layer between the gut lumen and the enterocytes. In some studies of healthy horses, Verrucomicrobia have been reported to account for up to 40% of the fecal microbiota [3]. In this study, Verrucomicrobia accounted for a much smaller percentage (i.e., 2.6–5.4%) of the total fecal microbiota at the four baseline sampling points, but even further decreased on Day 3 in fecal samples. This trend to decline after antibiotic use has been previously noted in both dogs and humans, and is thought to play a role in the loss of the intestinal barrier function in colitis.

All five of the horses in this study were PCR positive for *Salmonella* in their cecal content, but not in fecal samples, after metronidazole administration. Although not routinely tested for *Salmonella* before this study, the horses had never previously displayed symptoms of GIT disease. Identification of 3 serovars of *Salmonella* from the horses lends evidence against a herd outbreak of infectious *Salmonella*, which would typically include one serovar. All horses, except one, reverted to negative PCR status after the discontinuation of the metronidazole by the final study time point. Also, all horses were PCR negative at the 4 pre-treatment time points in both sample locations and remained negative in the fecal samples. Thus, the authors suggest that the metronidazole induced a degree of dysbiosis, which resulted in expansion of this enteric pathogen in the cecum, but not in the distal hindgut.

This study employed GC-MS methods to identify and quantitate changes in the end products of metabolism in the cecal and fecal samples before and after metronidazole administration. Of these 2 sample types, only the fecal metabolites were signficantly altered from baseline after adjustments for multiple comparisons were made. The fecal metabolites represented diverse metabolic pathways, such as nucleic acid, amino acid, carbohydrate, lipid and cofactor and vitamin metabolism. All significant metabolites belonging to the amino acid group were increased on Day 3. This could result from alterations in the commensal bacteria amd their role in the absorption or synthesis of these amino acids. Similar trends have been reported in dogs and humans with inflammatory bowel disease [33, 34]. Ribose, a metabolite of the pentose phosphate pathway in carbohydrate metabolism, was significantly elevated on Day 3, consistent with an oxidative stress response noted in the metabolomic profiles dogs with gastrointestintal disease [33]. Similarly, decreases in tocopherols, analogs of the anti-oxidant vitamin E, have been reported in horses suffereing from obesity [35].

Although metronidazole is reportedly safe to use in equine patients, the horses in this study appeared to develop early indications of colitis despite having normal feces. AAD is poorly defined in the veterinary literature in regards to the number or character of abnormal stools, but is generally regarded as a temporal association with the initiation or discontinuation of an antimicrobial agent and the development of diarrhea [9]. While diarrhea is characteristically considered pathognomonic for colitis, most horses exhibit prodromal symptoms associated with the gastrointestinal tract before diarrhea is clinically manifested. These symptoms often include inappetance, malaise, fever, and behavioral expressions of abdominal pain that precede the development of diarrhea. The clinical impression is that AAD is often acute in onset, occurring rapidly after the initiation of antimicrobial therapy. In horses, one study reported that the average time for development of diarrhea was 3.4 days after antibiotic administration (range: 1-11days) [36]. In humans, there is also evidence for the development of diarrhea associated with antibiotic discontinuation. This may have played a role in the death of Horse 3 on Day 14.

In this study, metronidazole decreased the diversity and altered the bacterial composition of cecal content and fecal samples. Subsequent functional alterations of the microbiome were reflected in the metabolite profile of the fecal samples. The timing of these changes coincides with the development of symptoms of GIT disease in these horses. Antibiotic administration, including metronidazole, is recognized as a risk factor for the development of diarrhea in species, such as humans, dogs, cats, and horses [3, 37–40].

## Supporting information

S1 File FAMEs, quality control samples used in GL-MS chromatography. (DOCX)

S1 Table The abundance of cecal taxa at the phylum level by study day. (XLSX)

S2 Table The abundance of cecal taxa at the family level by study day. (XLSX)

S3 Table The abundance of fecal taxa at the phylum level by study day. (XLSX)

S4 Table The abundance of fecal taxa at the family level by study day. (XLSX)

S5 Table Significant fecal metabolites by study day and KEG pathway. (XLSX)

S1 Fig Fecal metabolites in pathways of amino acid metabolism. (TIF)

S2 Fig Fecal metabolites in pathways of nucleic acid metabolism. (TIF)

S3 Fig Fecal metabolites in pathways of carbohydrate metabolism. (TIF)

S4 Fig Fecal metabolites in pathways of lipid metabolism. (TIF)

S5 Fig Fecal metabolites in pathways of the metabolism of cofactors and vitamins. (TIF)
