# Supplementary material for: The cecal and fecal microbiomes and metabolomes of horses before and after metronidazole administration
Source: PLoS One. 2020 May 22;15(5):e0232905. doi: 10.1371/journal.pone.0232905 (PMC7244109; doi:10.1371/journal.pone.0232905)
Supplement: S1 File — (DOCX) [file pone.0232905.s001.docx]

Supplemental File 1.

| **FAME**   \| **Compound** \| **Vendor** \| **Product Number** \| \| --- \| --- \| --- \| \| Methyl caprylate/octanoate (C08) \| Sigma \| 1424255 \| \| Methyl pelargonate/nonanoate (C09) \| Sigma \| 76368 \| \| Methyl caprate/decanoate (C10) \| Sigma \| 1424233 \| \| Methyl laurate/dodecanoate (C12) \| Sigma \| 234591 \| \| Methyl myristate/tetradecanoate (C14) \| Sigma \| M3378 \| \| Methyl palmitate/hexadecanoate (C16) \| Sigma \| P5177 \| \| Methyl stearate/octadecanoate (C18) \| Sigma \| S5376 \| \| Methyl arachidate/icosanoate (C20) \| Sigma \| A3881 \| \| Methyl behenate/docosanoate (C22) \| Sigma \| B3271 \| \| Methyl tetracosanoate (C24) \| Sigma \| L6766 \| \| Methyl hexacosanoate (C26) \| Sigma \| H6389 \| \| Methyl octacosanoate (C28) \| Sigma \| 74701 \| \| Methyl triacontanoate (C30) \| Sigma \| COM448666712 \| |
| --- | --- | --- | --- | --- | --- | --- | --- | --- | --- | --- | --- | --- | --- | --- | --- | --- | --- | --- | --- | --- | --- | --- | --- | --- | --- | --- | --- | --- | --- | --- | --- | --- | --- | --- | --- | --- | --- | --- | --- | --- | --- | --- |

|  |  |  |  |
| --- | --- | --- | --- |
|  |  |  |  |
|  |  |  |  |
|  |  |  |  |
|  |  |  |  |
|  |  |  |  |
|  |  |  |  |
|  |  |  |  |
|  |  |  |  |
|  |  |  |  |
|  |  |  |  |
|  |  |  |  |
|  |  |  |  |
|  |  |  |  |
|  |  |  |  |
|  |  |  |  |
|  |  |  |  |
|  |  |  |  |
|  |  |  |  |
|  |  |  |  |
| **Quality Control Mixples** |  |  |  |

| **Compound** | **Retention Time (s)** | **Solvent** | **Weight** | **Vendor** | **Product Number** |
| --- | --- | --- | --- | --- | --- |
| pyruvate | 6.74 | Water | 10 | Sigma | P2256 |
| alanine | 7.53 | Water | 10 | Sigma | A7627 |
| valine | 9.16 | Water | 10 | Sigma | V0500 |
| serine | 9.74 | Water | 10 | Sigma | S4500 |
| nicotinic acid | 10.258 | Water | 10 | Sigma | N4126 |
| succinic acid | 10.52 | Water | 10 | Sigma | S3674 |
| methionine | 11.82 | Water | 20 | Sigma | M9625 |
| *aspartic acid | 12 | Solution A | 20 | Sigma | A9256 |
| 4-hydroxyproline | 12.62 | Water | 10 | Sigma | H54409 |
| salicylic acid | 13.089 | Water | 10 | Sigma | S5922 |
| glutamic acid | 13.37 | Solution A | 10 | Sigma | G1251 |
| creatinine | 13.66 | Water | 10 | Sigma | C4255 |
| alpha ketoglutaric acid | 13.85 | Water | 10 | Sigma | 75890 |
| n-acetylaspartic acid | 14.8 | Water | 10 | Sigma | S783838 |
| asparagine | 14.97 | Water | 10 | Sigma | A0884 |
| putrescine | 15.77 | Water | 10 | Sigma | P7505 |
| shikimic acid | 16.493 | Water | 10 | Sigma | S5375 |
| citric acid | 16.63 | Water | 10 | Sigma | 251275 |
| lysine | 16.975 | Water | 10 | Sigma | L5501 |
| d-(+)-glucose | 17.47 | Water | 10 | Sigma | G8270 |
| glucose-6-phosphate | 21.381 | Water | 10 | Sigma | G7879 |
| arachidic acid | 22.354 | Chloroform | 10 | Sigma | A3631 |
| serotonin | 22.51 | Water | 10 | Sigma | H9523 |
| adenosine | 23.862 | Solution A | 10 | Sigma | A9251 |
| sucrose | 23.95 | Water | 10 | Sigma | S7903 |
| chlorogenic acid | 26.39 | Methanol | 10 | Sigma | C3878 |
| alpha tocopherol | 27.397 | Chloroform | 10 | Sigma | T3251 |
| cholesterol | 27.528 | Chloroform | 10 | Sigma | C8667 |

**Directions for preparing the QC samples**

1. Prepare 500 ml solution A as a mixture of H2O: methanol : isopropyl alcohol (1:2.5:1, v/v/v).Purge solution with nitrogen gas for 5 minutes to remove dissolved oxygen, for example using a pasteur pipet attached to a polypropylene line connected to a nitrogen gas tank.

2. Weigh standards into a glass vial according to Table 1 to 0.1 mg accuracy. Dissolve standards in (1 ml to 1.5 ml) appropriate solvents as listed in Table 1 in 2 ml Eppendorf tubes. Vortex mix for 10 s. Dissolve aspartic acid, glutamic acid, and adenosine to 1 mg/mL (20 mL, 10 mL, and 10 mL respectively) in solution A. These three compounds are difficult to dissolve at 10 mg/mL. For dissolving aspartic, add 5 uL at a time of 0.2 M NaOH after dissolving to 1 mg/mL.

3. Add approximately 25 mL of solution A to a 250 mL volumetric flask with glass stopper.

4. Transfer all the standard solutions quantitatively to 250 mL volumetric flask and adjust the volume by filling 250 mL volumetric flask with solution A to the calibration mark

5. Mix the QC mix for about 30 minutes (or more) to completely dissolve all compounds. This is the stock solution. Concentration of the various compounds in the stock solution is 40 μg/mL. This solution is stored in a refrigerator. The solution has a shelf life of 2 months

6. To make a working Quality Control mix: Dilute 2.5 mL stock solution to 10 mL with solution A to obtain a working concentration of 10 μg/mL. This standard solution is kept in the refrigerator

Six-point QC mix samples are pipetted from working QC mix into 2 ml Eppendorf vials.

QC6: 50 μL aliquot → 500 ng/compound in vial

QC5: 25 μL aliquot → 250 ng/compound in vial

QC4: 10 μL aliquot → 100 ng/compound in vial

QC3: 5 μL aliquot → 50 ng/compound in vial

QC2: 2.5 μL aliquot → 25 ng/compound in vial

QC1: 1.0 μL aliquot → 10 ng/compound in vial

7. All aliquots are taken when the solutions are at room temperature and standard solutions should be inspected before aliquots are taken so there is no precipitation.

8. QC mixes are dried down, derivatized and analyzed according to Basic Protocol 1. If 100 ul reagent volumes are used for derivatization, and 1 ul is injected (for GCquadrupole MS), then QC4 would represent ca. 1 ng injected onto the column per compound

9. Six point calibration standards are visually evaluated before and after each GC- MS analysis
