# Supplementary figures and images for: The cecal and fecal microbiomes and metabolomes of horses before and after metronidazole administration
Source: PLoS One. 2020 May 22;15(5):e0232905. doi: 10.1371/journal.pone.0232905 (PMC7244109; doi:10.1371/journal.pone.0232905)

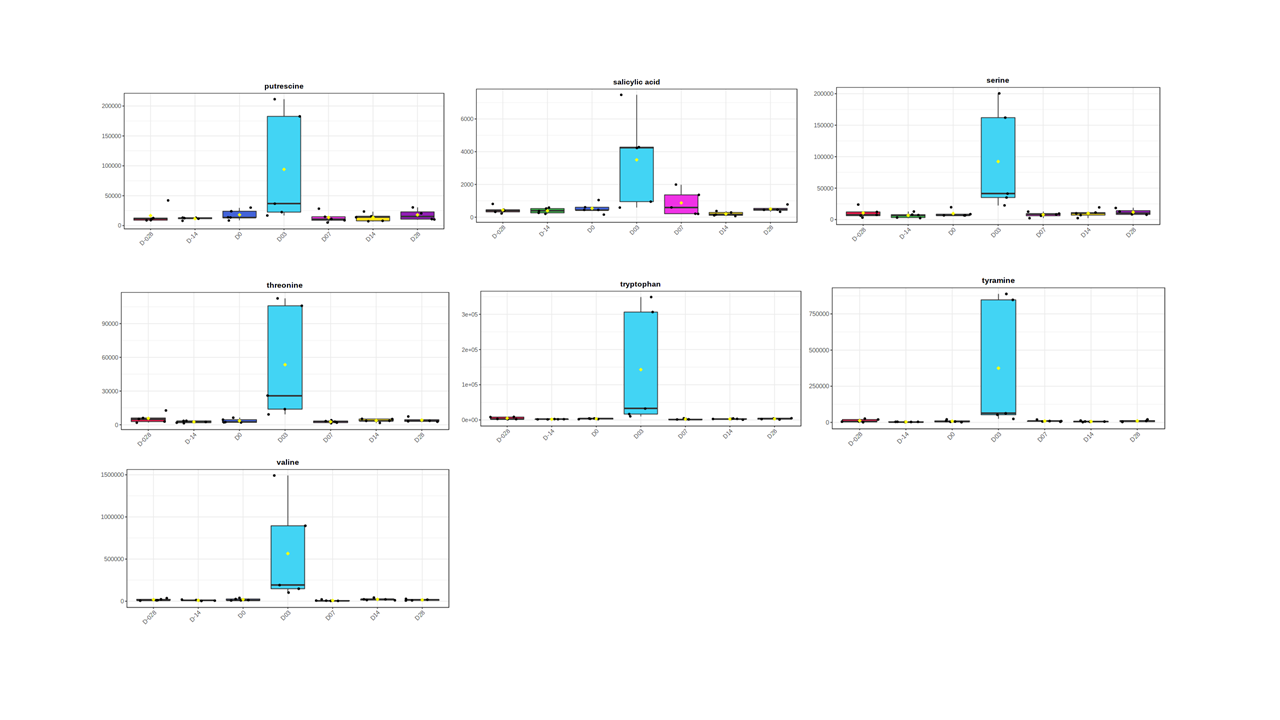

Supplement: S1 Fig — (TIF) [file pone.0232905.s007.tif]

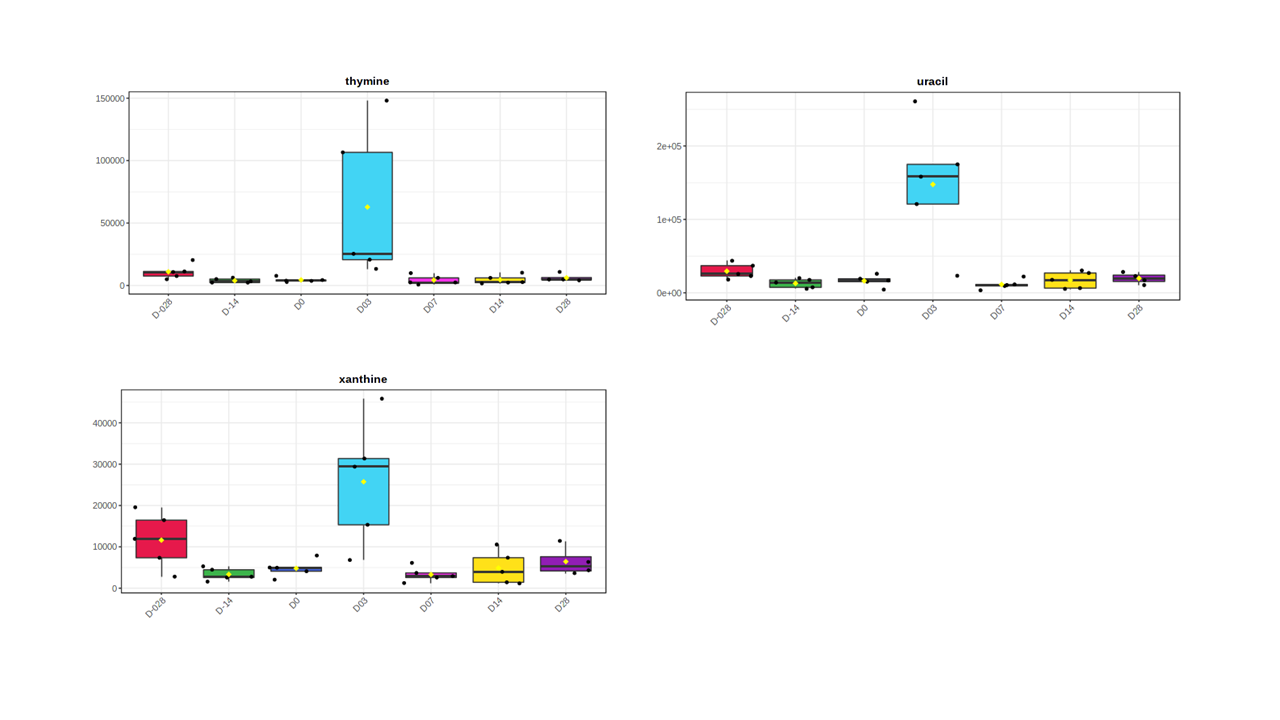

Supplement: S2 Fig — (TIF) [file pone.0232905.s008.tif]

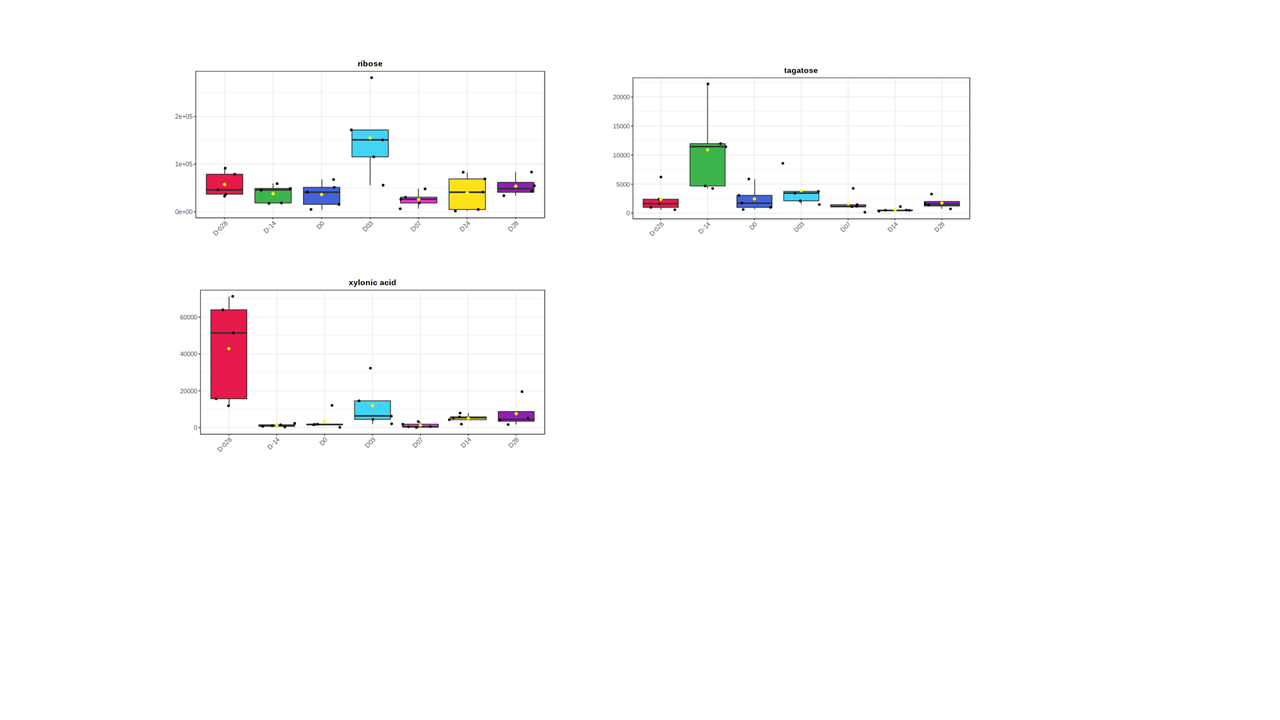

Supplement: S3 Fig — (TIF) [file pone.0232905.s009.tif]

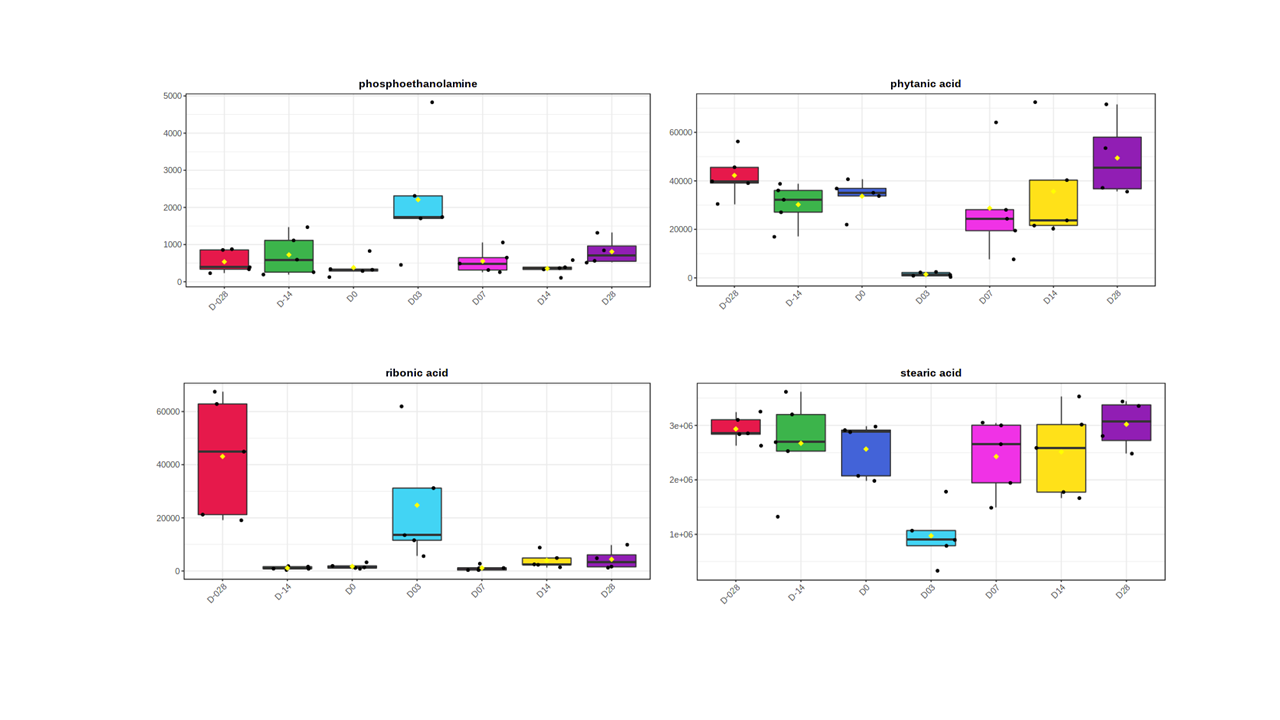

Supplement: S4 Fig — (TIF) [file pone.0232905.s010.tif]

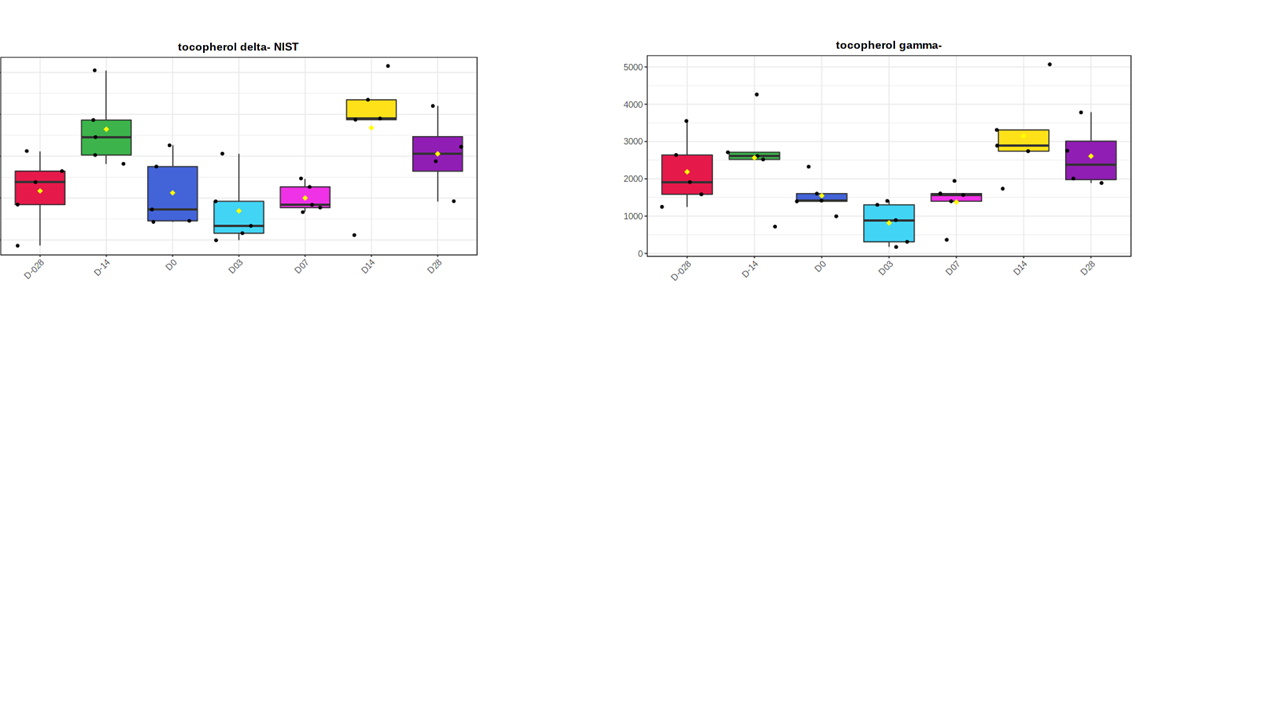

Supplement: S5 Fig — (TIF) [file pone.0232905.s011.tif]
